# An Enhanced Adaptive Kalman Filter for Multibody Model Observation

**DOI:** 10.3390/s25072218

**Published:** 2025-04-01

**Authors:** Antonio J. Rodríguez, Emilio Sanjurjo, Miguel Ángel Naya

**Affiliations:** Laboratory of Mechanical Engineering, CITENI, Campus Industrial de Ferrol, Universidade da Coruña, 15403 Ferrol, Spain; antonio.rodriguez.gonzalez@udc.es (A.J.R.); emilio.sanjurjo@udc.es (E.S.)

**Keywords:** virtual sensing, digital twin, force estimation, Kalman filter, noise modeling, colored noise, adaptive Kalman filter, shaping filter, multibody dynamics

## Abstract

The topic of state estimation using multibody models combined with Kalman filters has been an active field of research for more than 15 years now. Through state estimation, virtual sensors can be used to increase the knowledge of a system, measuring variables that cannot be obtained through conventional sensors. This is useful for control purposes or updating the state of a digital twin of a system. One of the most tricky questions with the different approaches tested in the literature is the parameter tuning of the filters, in particular, the covariance matrix of the plant noise. This work presents a new method which includes a shaping filter to whiten the plant noise combined with an adaptive algorithm to adjust the plant noise parameters. This new method is tested and compared with methods already described in the literature using the *three-simulation method*. The new method is at least as accurate as the best hand-tuned filters in most of the situations evaluated, and improves the accuracy of previously presented adaptive methods. All the methods and mechanisms tested in this paper are available in an open source library written in matlab called MBDE.

## 1. Introduction

The Kalman filter is an algorithm that provides an estimate of unknown states of a system [1,2]. Its operation is based on combining a model of the system with measurements over time of system variables provided by sensors. The filter takes into account the noise from the sensors and the system model, assuming it to be white noise, meaning it follows a normal distribution with zero mean. When dealing with linear systems and white noise, the observation provided by the filter is statistically optimal in terms of minimum mean square error. It has been applied in multiple fields, especially for control algorithms as virtual sensors for providing additional information of the system [3,4,5,6,7]. Virtual sensors can also be used in other use cases to give more insight about the system under study, such as for digital twin applications.

In its discrete version, which is the most used in practice, the filter consists of two stages. The first is the prediction stage, where a value for the states of the system is obtained from the previous states, the system model, and the noise. In the next stage, the states are corrected by adding the innovation to them. The innovation is the mismatch between the expected sensor measurements and the actual sensor data. The statistical properties of the innovation sequence play a significant role in several techniques described hereafter.

To extend its use to non-linear systems, variants of the Kalman filter have been developed. The most popular is the Extended Kalman filter [8,9,10], which makes a first-order Taylor series expansion of the system equations around the value of the a priori states of the system. For this reason, it uses the Jacobian of the system model as a transition matrix. Other variants have also been developed, such as filters using Sigma Points [11], which are used instead of a Taylor series expansion. Sigma points are sets of points deterministically obtained around the set of system states that have the same mean and covariance. Probably the most popular of these filters is the Unscented Kalman Filter [12].

In recent years, several applications of the Kalman filter to mechanisms modeled using multibody dynamic techniques have been presented [13]. Among the various filters developed, the errorEKF has shown more precise results than those obtained with other algorithms and does not consume a high computational load [14]. The errorEKF is an indirect estimator, meaning that the states are not the variables of interest, but the errors made by the multibody model, which is then corrected. This design allows us to use any multibody formulation.

In filter design, a difficulty lies in adequately characterizing the noise of the system, especially when it can vary during operation. This noise usually comes from modeling errors or unknown inputs, and therefore it is commonly difficult to characterize. In Kalman filters, the noise is unknown but it is assumed to be additive white Gaussian noise. If all of these assumptions are fulfilled, the noise can be characterized by its covariance matrix, and the Kalman filter will be an optimal estimator for a linear system.

In most applications, the values of the covariance matrix are usually obtained following a trial-and-error procedure [15,16,17,18], which increases the complexity of the filter implementation. In cases where the noise is white, but its covariance matrix is unknown or variable in time, adaptive filters may be employed to estimate the covariance matrix of the system noise. Two fundamental families of adaptive filters have been described in the literature: those that obtain their estimation from a bank of models, known as multiple-model-based adaptive estimation (MMAE), and those that analyze the innovation of the filter to estimate the noise of the system, known as innovation-based adaptive estimation (IAE) [19,20].

The MMAEs calculate the probability that each of various models corresponds to the problem in question based on sensor data. This involves calculating several models of the system in parallel, which can be computationally heavy if used with multibody models, limiting its use to applications where real-time execution is not necessary.

IAE filters assume the hypothesis that the innovation will be white noise if the filter has the appropriate values of the covariance matrices. Their computational load is not problematic in real-time applications and, therefore, they are more suitable for use with multibody models of the system. The principle of operation of this type of filter is based on the study of the innovation sequence of the Kalman filter; if the mean and covariance of the innovation do not have white noise properties, it can be concluded that the filter is not working correctly, possibly due to incorrect modeling of system noises [21,22]. There are multiple alternatives within this family of filters based on different principles. In [23], a comparative study of the different existing adaptive methods is presented. The most popular are maximum likelihood, covariance matching, and variational Bayesian.

Initially, the maximum likelihood (ML) criterion was used in inertial navigation systems where a Kalman filter was employed to cover the lack of GPS signal [19]. In ML, the goal is to obtain the value of the noise covariances that maximize the probability that a sequence of system innovation is minimal. The ML considers the innovation values obtained in a time window. This adds the window size as a new parameter to adjust the filter. If the window is very large, the estimate converges to the true value of the covariance matrices but will react very slowly in the case of a change in operating conditions. On the other hand, if small sample sizes are used (overtrained), an optimal value of the covariance matrices may not be reached.

Among the covariance matching methods, the Sage–Husa method [24] stands out, which obtains an optimal estimate of the covariance matrix by maximizing the unconditioned density function of the state variance matrix. This estimate provides the optimal covariance matrix used in that time step; hence, it is called maximum a posteriori probability (MAP). This method optimizes the covariance at each time step and does not employ a sequence of innovation data. However, the estimation of one time step is based on the previous step. This has given rise to methods that use a forgetting factor to regulate the contribution of the last time step compared to the historical one [25].

Both covariance matching and ML methods provide a determined value to the noise covariance matrices. A third method, Variational Bayesian (VB) [26], characterizes these matrices using a probability density function. This method assumes that the covariance matrix is described by an inverse gamma distribution. To correctly estimate the properties of this distribution, the vector of filter states is augmented with the statistical parameters that define the distribution. The advantage of this method is that it mitigates the effect of overtrained filters [26]. However, VB has been developed assuming that the covariance matrix of the system is known, so it can only be used to estimate the covariances of the measurement noises (from the sensors). This makes it less interesting when the noise of the system is the target of the characterization.

In [27], the design of an adaptive filter for the EKF error with force estimation has been presented. This article presents the development necessary to apply the ML method with an extended indirect Kalman filter based on a multibody model of the system.

Previous methods are suitable for Gaussian white noises. In cases where the noise is not uncorrelated, it is possible to resort to the shaping filtering technique, which consists of increasing the states of the system with the noise and modeling it. It is common to use a Markov model powered by white noise [28]. The Markov model involves a time window of data to determine the autocorrelation time of the innovation sequence. In [13], an application of this technique to an extended observer based on multibody models was shown. The use of this technique showed an improvement in the innovation sequence with more uniform behavior at all frequencies, that is, closer to white noise. This results in more accurate filter estimates. Furthermore, if the observer already has a white innovation sequence, the inclusion of a shaping filter has no influence on the results.

The aim of this work is to increase the accuracy of the state estimators including a continuous and on-line characterization of the plant noise. This implies that the filter will automatically adapt its behavior to changes in the plant, and it also simplifies the implementation of Kalman filters in real applications. To achieve this goal, this work proposes the combination of the shaping filter with an adaptive filter to achieve an enhanced characterization of the noise. Adaptive filters assume that noise is white. However, when the noise of the system is completely unknown, it is not advisable to rule out the possibility that the noise is colored. The present work attempts to address this combination in observers based on a multibody model in order to adequately characterize the noise of the system. Along with the development of the equations to combine both techniques, another difficulty arises from the use of time windows in both the shaping filter and the adaptive filter ML method. Hence, it is also of interest to obtain guidance on them to achieve an optimal characterization of the system noise. The use of this technique, with proper guidance on window size, allows noise characterization to be automated (previously performed through trial and error), leading to more accurate estimations and to systems that can automatically adapt to changing conditions.

## 2. Materials and Methods

To carry out this work, the *three-simulation method* has been used, in combination with the MBDE library. The MBDE library is a toolbox written in Matlab intended for the development of state estimators based on multibody models. This library is released under a GPL license and can be downloaded from https://github.com/MBDS/mbde-matlab (accessed on 27 March 2025). In the *three-simulation method*, a first simulation is used instead of the real mechanism. This allows access to all the information about the kinematic and dynamic behavior of the mechanism that is used as a reference (ground truth).

To evaluate the performance of the proposed solution, this work addresses the problem of estimating the states of the system in terms of position, velocity and acceleration, together with the forces (or inputs) that produce the observed dynamics, using a minimal set of physical sensors. It is of particular interest to evaluate the accuracy obtained in the states or forces that are not directly measured, since those variables are the ones that can be used as virtual sensors for increasing the knowledge of the state of the system.

**Figure 1:**
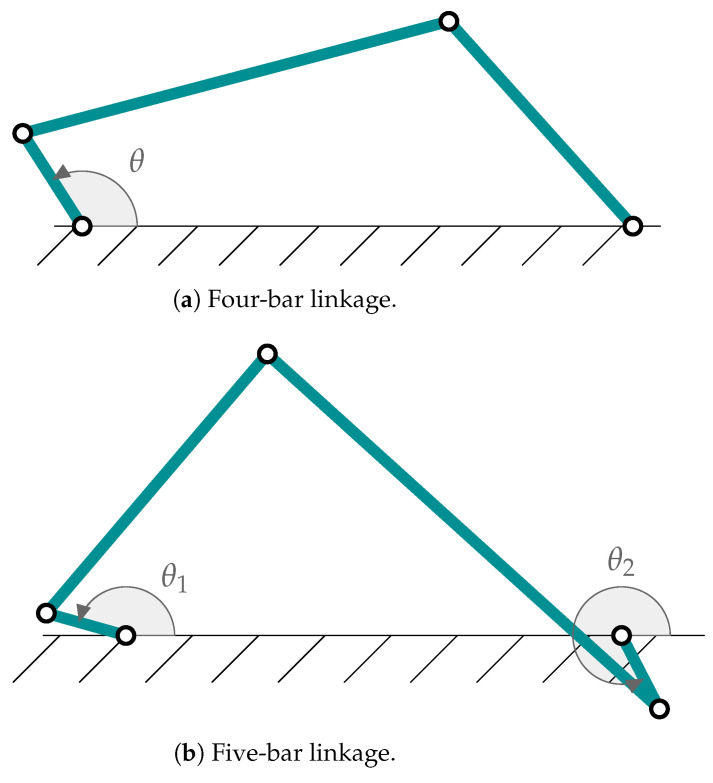
Mechanisms employed in this work.

In the present work, as in previous ones [13,14,27], tests have been carried out with two types of mechanisms: an articulated quadrilateral and a five-bar linkage, which have been represented in Figure 1. The characteristic magnitudes of each one are found in Table 1 and Table 2. These mechanisms have been chosen for simplicity and to take into account the effect of several degrees of freedom.

The readings of the different sensors considered are obtained from this model, as would be done in a real mechanism. White noise is added to these measurements to model the characteristic noise of the various types of sensors. The seed for the pseudorandom number generator used to generate the noise of the sensors is the same in all the tests, so the results of the simulations are deterministic, and the differences between tests depend only on the methods or parameters used. A second simulation of the mechanism is used as a model. This model tries to reflect the discrepancies that usually exist in the modeling of a real mechanism (differences in the characteristic parameters of the elements such as masses, lengths, inertias or in the torques such as the absence of friction forces; differences in the modeling of the forces applied to exteriors). The errors that have been introduced in this model are a difference of 1 m/s^2^ in the value of gravity and that the initial position of the crank differs by pi/16 rad with respect to that of the real mechanism.

As expected, the behavior of this model will not be identical to the first. The third simulation, the observer, will be responsible for the estimation of the real mechanism by combining the data from the sensors and the model with errors. The discrepancy between the real model and the observer model is characterized by a noise model. In this work, we explore an algorithm based on an adaptive Kalman filter with a shaping filter to approximate the best parameters for this plant noise during the execution of the observer, so accurate parameter tuning is not required, and the observer can adapt its behavior if the conditions change.

**Figure 2:**
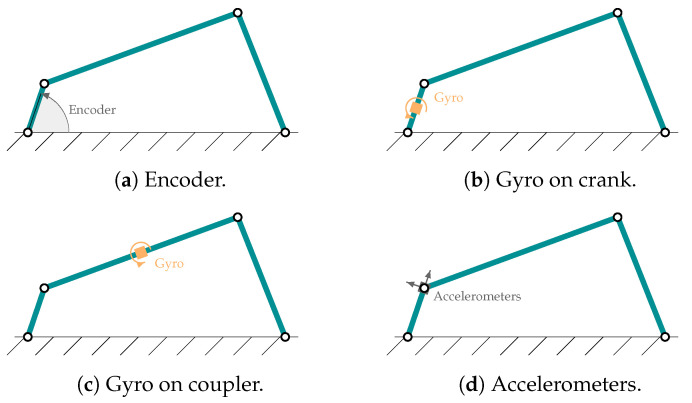
Sensor configurations considered in the four-bar linkage.

To verify the accuracy of this new algorithm, various sensors and combinations of sensors have been used to determine their influence on the results. The same combinations have been used as in previous works, as detailed in Figure 2. Specifically, for the four-bar linkage, the following sensors have been used: (a) an encoder installed on the crank, coinciding with the degree of freedom of the mechanism, (b) a gyroscope on the crank that measures its angular velocity, (c) a gyroscope in the mechanism coupler and (d) a biaxial accelerometer at the end of the crank with the axes aligned so as to capture the longitudinal and transverse accelerations with respect to it. In the case of the five-bar linkage, the same sensors have been used but duplicated since it has two degrees of freedom. The modeling of the accelerometers and gyroscopic sensors is detailed in [14], and their characteristics are shown in Table 3. These noise levels are compatible with low-cost sensors.

### 2.1. Multibody Formulation

Multibody system dynamics studies the dynamics of systems composed of several bodies, rigid and/or flexible, joined in such a way that some movements are allowed while others are prevented. The rigid nature of the bodies and the movement limitations introduced in the joints are modeled by algebraic equations called constraint equations. The union of the dynamic and constraint equations gives rise to the following system of Differential Algebraic Equations in which q is a set of *n* generalized coordinates [29]:

(1a) $$\begin{array}{cc} M\ddot{q}+\Phi_{q}^{T}\lambda & =Q \end{array}$$

(1b) $$\begin{array}{cc} \Phi \left(q,t\right) & =0 \end{array}$$

where M is the mass matrix of the system, $\ddot{q}$ the accelerations of the natural coordinates, Φ the constraints vector, $\Phi_{q}$ the Jacobian matrix of the constraint equations with respect to the generalized coordinates q, Q the generalized forces, and $\lambda^{*}$ the Lagrange multipliers [30]. The forces due to the imposition of the constraints are represented by the term $\Phi_{q}^{T}\lambda$.

Various formalisms have been developed to solve this set of equations. In this case, we used the matrix R method [29], although the indirect methods of observers based on the errorEKF, which are the ones used in this work, can be used with any other multibody formulation [14]. Matrix R formulation is briefly described hereafter for the convenience of the reader. For a more exhaustive explanation of the method, refer to [29].

If all the constraints are scleronomous, the following velocity transformation can be written:

(2) $$\dot{q}=R\dot{z}$$

where $\dot{q}$ is the whole vector of velocities, $\dot{z}$ is the vector of independent velocities, and R is a matrix which provides the complete velocity vector when it is multiplied by the vector of independent velocities.

The time derivative of the previous equation can be written as:

(3) $$\ddot{q}=R\ddot{z}+\dot{R}\dot{z}$$

By using these transformations, the equations of motion can be rewritten in independent coordinates as follows:

(4) $$R^{T}MR\ddot{z}=R^{T}\left(Q-M\dot{R}\dot{z}\right)$$

This equation can then be integrated using any suitable integrator. In this case, the trapezoidal rule was used.

### 2.2. State and Input Observer

The state estimator developed in this work is based on the Kalman filter. Although the Kalman filter is originally developed for linear systems, it has been extended for non-linear systems (such as multibody models), resulting in the well-known Extended Kalman filter (EKF). If the filter is expressed in discrete form, the estimation process can be divided in two stages: prediction and correction stage.

The prediction phase of the extended Kalman filter (EKF) for a time step *k* is performed following

(5) $$\begin{array}{ccc} {\hat{x}}_{k}^{-} & = & f({\hat{x}}_{k-1}^{+}) \end{array}$$

(6) $$\begin{array}{ccc} P_{k}^{-} & = & f_{x}P_{k-1}^{+}{f_{x}}^{T}+\Sigma^{P} \end{array}$$

where $\hat{x}$ is the vector of states of the estimator, P is the covariance matrix of the estimation error, f(·) is the non-linear function which represents the system behavior, $f_{x}$ is its Jacobian matrix with respect to the states, $\Sigma^{P}$ is the plant covariance matrix and the superindices ‘−’ and ’+’ mean apriori (before correcting the states with the sensor readings) and aposteriori (after fusing the sensor readings and the model predictions), respectively. The correction phase consists of incorporating the sensor readings in order to improve the model prediction, following

(7) $$\begin{array}{ccc} {\tilde{y}}_{k} & = & o_{k}-h({\hat{x}}_{k}^{-}) \end{array}$$

(8) $$\begin{array}{ccc} \Sigma_{k} & = & {h_{x}}_{k}P_{k}^{-}{h_{x}}_{k}^{T}+\Sigma^{S} \end{array}$$

(9) $$\begin{array}{ccc} K_{k} & = & P_{k}^{-}{h_{x}}_{k}^{T}\Sigma_{k}^{-1} \end{array}$$

(10) $$\begin{array}{ccc} {\hat{x}}_{k}^{+} & = & {\hat{x}}_{k}^{-}+K_{k}{\tilde{y}}_{k} \end{array}$$

(11) $$\begin{array}{ccc} P_{k}^{+} & = & \left(I-K_{k}{h_{x}}_{k}\right)P_{k}^{-} \end{array}$$

where ${\tilde{y}}_{k}$ is the innovation, which represents the information provided by the sensor readings o, which is not present in the a priori filter states, h(·) is the function relating the states and the measurements and $h_{x}$ its Jacobian matrix with respect to the states. $\Sigma_{k}$ is known as the innovation covariance matrix, and it represents the uncertainty in the system state projected through the sensor function and an additional Gaussian noise originating at the sensor itself, $\Sigma^{S}$. Small values of $\Sigma_{k}$ mean that the observation introduces useful information to constrain the estimation of the system state. By evaluating the Kalman gain K, the estimation of the mean and covariance are updated in Equations (10) and (11), respectively.

The innovation $\tilde{y}$ is an important indicator of the correctness of the filter; the innovations are Gaussian white noise when all the noise models are correct [22]. Hence, when significant patterns appear in the innovation sequence, inadequate noise characterization or important modeling errors should be suspected [21,22].

The methods considered in this work are based on the error-state Kalman filter, also known as indirect Kalman filter. In this type of filter, the states x are not the magnitudes of interest, but the errors of the system under study. In particular, the states of all the methods considered in this work are the position, velocity, and acceleration errors of the degrees of freedom of the multibody model, Δz, $\Delta \dot{z}$, and $\Delta \ddot{z}$, respectively, as follows:

(12) $$x={\left[\Delta z^{T},\Delta {\dot{z}}^{T},\Delta {\ddot{z}}^{T}\right]}^{T}$$

After each correction phase, the errors are used to correct the multibody model state. The estimated errors of the degrees of freedom and the kinematics equations are used to correct the position, velocity, and acceleration of the mechanism.

Once the positions and the velocities are corrected, their expected error is null. This does not mean that the correction is perfect, but that on average, the error should be zero. However, for the acceleration, the problem is different; if there is an unknown or an incorrect force, the model will also provide an incorrect acceleration. To solve this problem, the force required to correct the acceleration error can be calculated as:

(13) $$\Delta Q=R^{T}MR\Delta \ddot{z}$$

If ΔQ is added to the vector of generalized forces Q, the incorrect model would provide the corrected accelerations. After all these corrections are applied, the multibody model can be used as a virtual sensor, providing more information than the sensors used to correct its state. Since it is assumed that the force errors are persistent in time, the force errors estimated after the correction in one time step are applied for the prediction of the following time step, as follows:

(14) $$\Delta Q_{k}^{-}=\Delta Q_{k-1}^{+}$$

Therefore, since the positions and the velocities are corrected, and the forces are also corrected for the model to produce the correct accelerations, the expected value of the error of the model for the following time step is zero, so the equation of the propagation of the state of the Kalman filter, Equation (5), becomes ${\hat{x}}_{k}^{-}=0$.

This method with force estimation is explained more in depth in [14], where it is referred to as errorEKF_FE. For the sake of conciseness, in the present work, we will call this method errorEKF.

### 2.3. Noise Modeling

When developing a multibody model of a system, the main sources of error are expected to come from errors in forces. While the geometry of the system can be accurately measured during the modeling, other parameters such as the mass properties or the force models remain uncertain.

In a Kalman filter, the errors between the model and reality are considered through the plant noise. As stated before, in this work, the gravity force is modified so that there is an error in the force model. This error implies that there is an uncertainty in the system related to the force model. This error will result in a deviation in the behavior of the model with respect to the ground truth and, therefore, it should be reflected in the covariance matrix of the plant.

In addition, since the errors are only in the force model, the expected deviation at the plant comes from acceleration errors, which are then propagated through the integration of the multibody model to velocity and position level. Thus, assuming that the integration error is negligible compared to the acceleration error, the only part of the plant noise covariance matrix that has non-null terms is the part of its diagonal corresponding to the accelerations, as follows:

(15) $$\Sigma^{P}=\left[\begin{array}{ccc} 0 & 0 & 0\\ 0 & 0 & 0\\ 0 & 0 & \mathrm{diag}\left(\sigma_{1}\ldots \sigma_{g}\right) \end{array}\right]$$

where all the blocks are of size *g*, which is the number of degrees of freedom of the mechanism, and the term $\mathrm{diag}\left(\sigma_{1}\ldots \sigma_{g}\right)$ is a diagonal matrix with the variances of the acceleration noise of the plant for every degree of freedom. This approach simplifies the filter tuning, since only one term of the covariance matrix has to be set for every degree of freedom of the mechanism studied.

But even in this case, a certain level of experience is required to properly adjust the filter, and if the conditions vary, the settings will become incorrect. To improve this situation, an adaptive method for multibody dynamics simulations was proposed in a previous work [27]. This method was a big step forward towards the practical use of the state observers based on multibody models, since it allows an inexperienced user to implement the method, because filter tuning is no longer required. Moreover, the adaptive method, as its name suggests, can modify its settings on the fly depending on the conditions in which the mechanism is operating (for instance, in case of malfunction of the mechanism, or if the mechanism works in different conditions than those assumed by the model).

As explained in [27], the plant covariance matrix is estimated using the maximum likelihood algorithm (ML). Based on a sequence of data, this method seeks to obtain the covariance values which are more likely to produce such a sequence. The main advantage of this method is that it ensures a convergence to the true values of the covariance matrix. However, it requires a sliding window of data and, depending on the size of the window, the performance of the method can be affected, yielding to biased estimations.

For a general set of noises σ and observed data $\hat{z}=h({\hat{x}}_{k}^{-})$, the likelihood function describes the probability that the available observed measurements $\hat{z}$ could be observed using the given parameters σ [31]. These observations contain the same statistical information as the residuals of the Kalman filter, also known as innovations [32]. In an optimal filter, the innovation is assumed to be distributed as zero-mean, white Gaussian noise with covariance Σ (Equation (8)). Hence, the likelihood function *L* for a particular sequence of *N* samples can be defined as

(16) $$L\left(\sigma |\hat{z}\right)=\prod_{j=1}^{N}\frac{1}{\sqrt{{\left(2\pi \right)}^{m}\left|\Sigma_{j}\right|}}\exp^{-\frac{1}{2}{\tilde{y}}_{j}^{T}\Sigma_{j}^{-1}{\tilde{y}}_{j}}$$

where *m* is the number of measurements in the measurement vector $h({\hat{x}}_{k}^{-})$, *j* the counter of samples inside the sliding window, |·| the determinant operator, and exp the natural base. Note that σ are the covariances which are being estimated, $\tilde{y}$ is the innovation sequence for a window size of *N* samples, and Σ is the covariance matrix of the residuals of the Kalman filter. After some simplifications [19,27], the maximum likelihood condition can be derived:

(17) $$\sum_{j=j_{0}}^{k}ln\left|\Sigma_{j}\right|+\sum_{j=j_{0}}^{k}{\tilde{y}}_{j}^{T}\Sigma_{j}^{-1}{\tilde{y}}_{j}=min$$

where *k* is the time step in which the covariance matrix is being estimated and *j* the moving counter inside the estimating window.

The value of σ can be estimated by differentiating Equation (17) with respect to σ and setting the resultant equation to zero. Note that Σ depends on the covariance σ that is being estimated. Hence, minimizing Equation (17) is equivalent to finding the value of σ that will result in the smallest error norm [19]. For the particular case of estimating the σ related with the plant covariance matrix, it is assumed that the measurement covariance matrix is known and independent of σ [19]. This is true in most situations, since the measurements are independent of the plant errors and their covariance matrix can be obtained from the characteristics of the sensors. Relating Σ with $\Sigma^{P}$ and $\Sigma^{S}$ through Equations (6) and (9), the estimation of the plant covariance matrix can be obtained:

(18) $${{\hat{\Sigma }}^{P}}_{k}=\frac{1}{N}\sum_{j=j_{0}}^{k}\left[\Delta x_{j}\Delta x_{j}^{T}+P_{j}^{+}-{f_{x}}_{j}P_{j-1}^{+}{f_{x}}_{j}^{T}\right]$$

where $P_{j}^{+}-{f_{x}}_{j}P_{j-1}^{+}{f_{x}}_{j}^{T}$ reflects the variation in the covariances between time steps and $\Delta x_{k}$ is the state correction sequence. For further details, the reader is referred to [27].

However, the errorEKF algorithm with the plant noise tuned by trial-and-error behaves better than the adaptive method in several circumstances. This fact shows there is still room for improvement. The adaptive algorithm assumes that the noise is additive, white, and Gaussian. However, the noise in a multibody model usually comes from unmodeled forces (ideal joints, degradation of the real system, unknown friction coefficients, etc.). These errors usually have a low-frequency preponderance, that is, their effect produces a deviation of the model with respect to the reality that lasts several time steps. This is a problem for the adaptive algorithms which are based on the statistical properties of the innovation. However, this behavior can be improved using a shaping filter [21], which provides a plant noise with the adequate low frequencies when the shaping filter is fed with white noise, as follows:

(19) $$\omega_{k}=\psi_{k-1}\omega_{k-1}+\xi_{k-1}$$

where ω is a time-correlated noise, ξ is white Gaussian noise, and ψ is a weight which can take values from 0 to 1. The two extreme cases are ψ=0, when ω becomes a white noise, and ψ=1, when ω becomes a random walk.

In [13], the application of a shaping filter to errorEKF was explored using a state augmentation, that is, the values of the low-frequency noise *w* were added as states. In the present work, however, a slightly different approach is applied. The reason is that the design of the errorEKF somewhat includes this feature, since the force error estimation in one step is used in the prediction of the following step, as shown in Equation (14).

The underlying assumption behind this design is that the force errors are persistent, or, at least, their variation is slow compared to the length of a time step. Therefore, the forces are modeled as a random noise (equivalent to ψ=1 in Equation (19)). However, this model might not be always the best one. For instance, even a constant error force might have variable effect depending on the trajectory and the coordinates used to describe the multibody model. In addition, the force error might not be constant, nor have a slow variation.

For all these reasons, it is worth studying which is the best value of the weight ψ, which in the errorEKF will modulate the fraction of the error force estimation propagated to the following time step. Therefore, Equation (14) becomes:

(20) $$\Delta Q_{k}^{-}=\psi_{a}\Delta Q_{k-1}^{+}$$

where $\psi_{a}$ is a diagonal matrix containing the values of ψ corresponding to every degree of freedom at the acceleration level.

This approach showed a better behavior than that used in [13] when combined with the adaptive algorithm. Moreover, with this approach, the state is not augmented, thus saving computational cost.

When $\psi_{a}$ is properly adjusted, it enables better working of the adaptive algorithm, which assumes that the plant error is white noise. In this work, we studied the autocorrelation of the innovation sequence to set the proper value of $\psi_{a}$. In this case, a sliding window of a fixed size *n* is considered, and the autocorrelation of the innovation within this window is studied. Since the model in Equation (19) relates the value in one time step with the one in the previous time step, the relevant magnitude here is the autocorrelation with the lag of 1 time step, as follows:

(21) $$\rho_{y,y}\left(k,k-1\right)=\frac{\sum_{i=k-n+1}^{k}\left(y_{i}-\bar{y}\right)\;\cdot \;\left(y_{i-1}-\bar{y}\right)}{\sigma_{y}^{2}}$$

where $\rho_{y,y}\left(k,k-1\right)$ is the autocorrelation of the moving window of a component *y* of the innovation sequence $\tilde{y}$ for the time step *k*, and with a lag of 1 time step, $y_{i}$ is the component of the innovation in time step *i*, *n* is the size of the window, and $\bar{y}$ and $\sigma_{y}^{2}$ are the mean and variance of the component of the innovation sequence of the considered window, respectively.

The autocorrelation calculated in this way has one component for every sensor. However, the shaping filter described here is to model the plant, not the measurements. Therefore, in order to modify the plant, the autocorrelation of the innovation has to be projected over the states. In order to do that, the Kalman gain matrix is used, since it conveys the information from the measurements to the states. However, since the weight ψ has to be between 0 and 1, all the columns of the Kalman gain matrix are normalized dividing by the value of their 2 norm, as shown:

(22) $$K_{U}=\left[\begin{array}{cccc} \frac{k_{1}}{\left|\right|k_{1}\left|\right|} & \frac{k_{2}}{\left|\right|k_{2}\left|\right|} & \ldots & \frac{k_{l}}{\left|\right|k_{l}\left|\right|} \end{array}\right]$$

Then, the normalized Kalman gain matrix $K_{U}$ is used to update the values of ψ contained in matrix ψ:

(23) $$\psi_{k}=\psi_{k-1}+\mathrm{diag}\left(\frac{K_{U}\rho_{\tilde{y},\tilde{y}}\left(k,k-1\right)}{n}\right)$$

where $\rho_{\tilde{y},\tilde{y}}\left(k,k-1\right)$ is a vector containing the autocorrelation of all the components of the innovation, calculated as in Equation (21), and the operator diag() takes a vector and converts it to a diagonal matrix. In this way, the components of ψ increase their value when the innovation sequence is autocorrelated. The first third of the elements of ψ correspond to the position level of the degrees of freedom, the second third corresponds to the velocity, and the last third is $\psi_{a}$, the part used in Equation (14), which corresponds to the accelerations. This update is applied every time step, but the last *n* time steps are included in the sliding window used to calculate the autocorrelation, and therefore, the increment of ψ is divided by the size of the window *n* to avoid adding *n* times the contribution of each sample.

One of the consequences of this algorithm is that the sequence of innovation contained in the window of size *n* might be obtained with different values of ψ. Moreover, the innovation sequence has an intrinsic level of uncertainty. Therefore, it is not expected that the autocorrelation reaches a null value. In this work, a threshold for the autocorrelation of $\pm 1.96\;\cdot \;\sigma_{y}$ was used; if the autocovariance value of a component of the innovation is below this limit, the value of the corresponding value of ψ is not updated in Equation (23). The threshold used implies that a sequence of white noise will have a value below the selected threshold 95% of the time.

By the application of this strategy, the plant noise is whitened, and this should improve the accuracy of the adaptive algorithm. Therefore, by combining both techniques, an adaptive errorEKF with force estimation combined with a shaping filter is obtained. This will be referred to as AerrorEKF_SH in the rest of this document.

## 3. Results and Discussion

To verify the behavior of the new method presented here (an adaptive errorEKF with force estimation and a shaping filter, AerrorEKF_SH), the two benchmark mechanisms shown in Figure 1 were tested with this new formulation and compared with simpler formulations. The sensors considered in the experiments are the ones depicted in Figure 2, in particular, the errorEKF with force estimation (here named errorEKF), which is tuned by trial an error, and its adaptive version, but without the shaping filter (AerrorEKF). Both the adaptive part of the algorithm and the shaping filter require a moving window of data to reliably extract the relevant statistical information of the signals. The larger the window of data, the more accurate information can be achieved. However, if the mechanism changes its configuration, or the conditions in which it is operating, a large window will slow down the adaptation to the new set of conditions. Therefore, depending on the type of mechanism and the variability of the conditions of operation, there will be a window size that will be more adequate to achieve the best results.

### 3.1. Window Size Selection

The method presented in this work requires two parameters that should be set by the user: the size of the moving windows for the plant noise estimation and the weight of the shaping filter (ψ) estimation. As expressed in [27], the size of the window is dependent on the application. In order to provide a better insight on the effects of the window sizes on the estimations, this section compares the results of several simulations with different window sizes. The experiments are performed using the four-bar and five-bar linkage mechanisms (Figure 1) with all the sensor configurations considered in this work (Figure 2).

**Figure 3:**
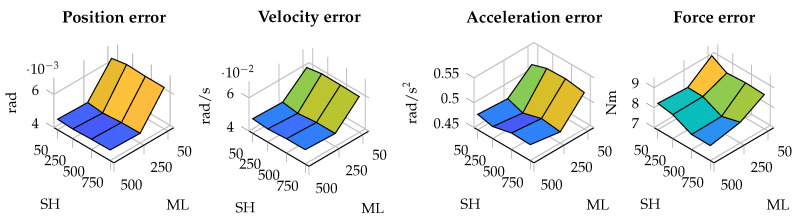
Estimation errors in the four-bar linkage with an enconder in the crank for different window sizes. The horizontal axis represents the window size used in the shaping filter (SH) and maximum likelihood algorithm (ML).

In terms of estimation errors, in most of the tested cases, the error is more affected by the size of the window for estimating the plant covariance matrix. The results in terms of estimation errors in the four-bar linkage with an encoder in the crank are shown in Figure 3. As can be seen, the error decreases as the window employed by the maximum-likelihood algorithm increases, while the size of the window for the shaping filter has less influence.

**Figure 4:**
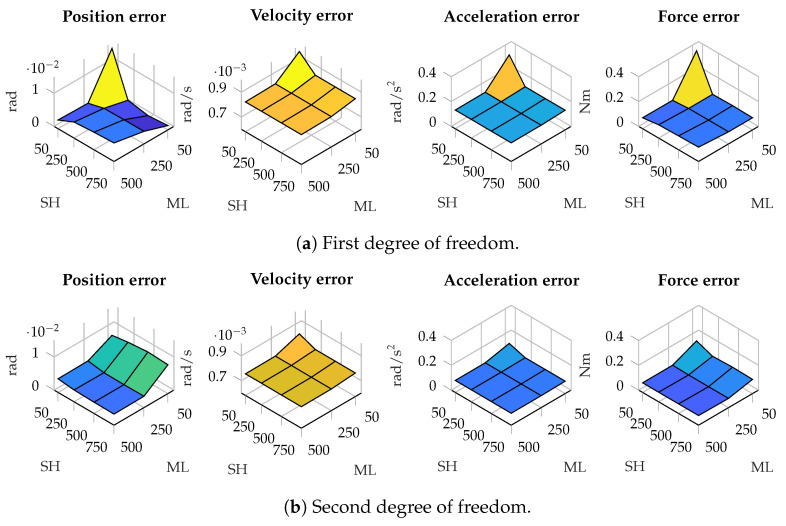
Estimation errors in the five-bar linkage with gyroscopes on both cranks for different window sizes. The horizontal axis represents the window size used in the shaping filter (SH) and maximum likelihood algorithm (ML).

There are scenarios where the window size of the shaping filter has more effect. As an example, Figure 4 presents the error in the estimations in position, velocity, acceleration and force in the five-bar linkage with gyroscopes on the cranks. As can be seen, in the second degree of freedom, the error decreases as the window employed by the maximum-likelihood algorithm increases, while the effect of the size of the window for the shaping filter has less influence. In the first degree-of-freedom, however, the window size of the shaping filter has higher relevance in the accuracy of the estimations: a small window size for the shaping filter, combined with a small window size for the maximum-likelihood algorithm, results in higher errors.

**Figure 5:**
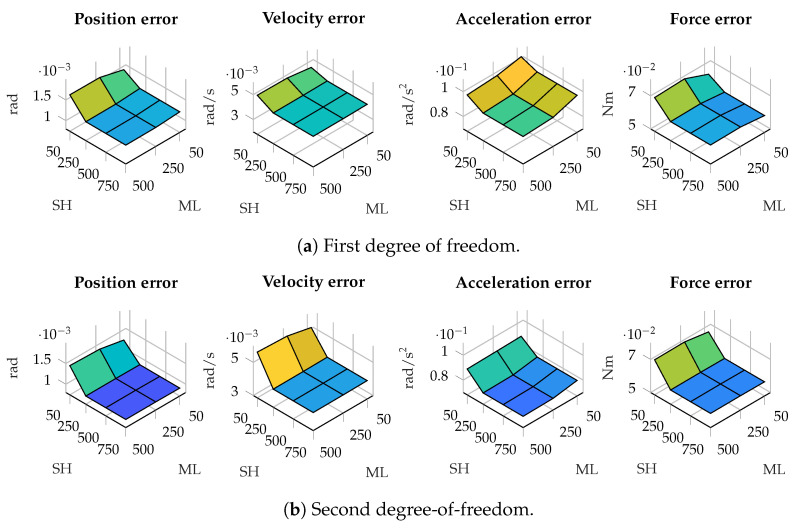
Estimation errors in the five-bar linkage with a pair of accelerometers on both cranks for different window sizes. The horizontal axis represents the window size used in the shaping filter (SH) and maximum likelihood algorithm (ML).

This behavior can also be seen in the five-bar linkage with a pair of accelerometers on each crank. The estimation errors in this scenario are shown in Figure 5. In this case, a window of small size in the shaping filter results in higher errors, in spite of the window size employed in the maximum-likelihood algorithm.

The explanation for these results can be related with the properties of the autocorrelation of the innovation. When the four-bar linkage is combined with an encoder on the crank, the innovation sequence of the conventional errorEKF is already close to white noise. Hence, the effect of the shaping filter is less relevant that the effect of the ML. However, in the case of the five-bar linkage with gyroscopes on the cranks, the innovation sequences are quite autocorrelated for both sensors, especially for the gyroscope placed on the first crank. Therefore, if the innovation of the system is autocorrelated, a shaping filter with a small window size does not effectively correct the autocorrelation of the innovation, yielding higher errors.

The autocorrelation of the innovation sequence for the four-bar linkage with an enconder on the crank is shown in Table 4. In this configuration, the innovation in the errorEKF can be considered white noise because of the value of its autocorrelation. Therefore, the innovation sequence used to estimate ψ is going to be close to white noise in spite of the window size selected. The most determinant factor in these situations is the window size employed for the estimation of the plant noise. In addition, the estimated plant noise will also affect the minimum autocorrelation that can be achieved in the innovation sequence.

When simulating the five-bar linkage with gyroscopes on the cranks, the innovation sequences of both sensors in the errorEKF are autocorrelated, especially in the gyroscope of the first crank. In this situation, the window size selected for the shaping filter has an influence on the accuracy of the results and in the whiteness of the innovations. As can be seen in Table 5, the simulations with the smaller window size lead to innovation sequences which are not as white as expected. This turns into higher errors in the estimations, as shown in Figure 4.

**Figure 6:**
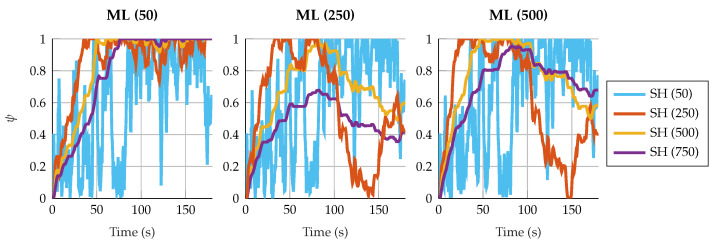
Estimated value of ψ in the four-bar linkage with an encoder on the crank. The sliding window size for the maximum-likelihood algorithm varies from 50 to 500 samples. For the shaping filter, the window varies from 50 to 750 samples.

**Figure 7:**
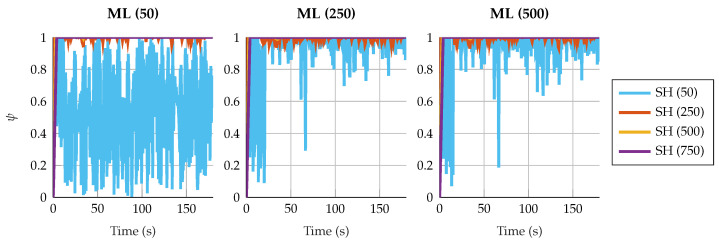
Estimated value of ψ in the five-bar linkage with a gyroscope on each crank. The sliding window size for the maximum-likelihood algorithm varies from 50 to 500 samples. For the shaping filter, the window varies from 50 to 750 samples.

This behavior is also related with the estimation of ψ. With small window sizes for the shaping filters, the value of ψ fluctuates rapidly. Figure 6 and Figure 7 show the estimation of ψ under different window sizes for the four-bar linkage with an encoder on the crank and the five-bar linkage with a gyroscope on each crank. In cases where the innovation is already close to white noise, as in the case of the four-bar linkage with an encoder on the crank, these fluctuations do not affect in the accuracy of the results. However, when the five-bar linkage is configured with two gyroscopes in the crank, the innovation is still autocorrelated due to the strong fluctuations of ψ. When the window size of the shaping filter increases, the value of ψ is more stable and the Aerror_SH is capable of correcting the autocorrelation of the innovation sequence, improving the accuracy of the results.

**Figure 8:**
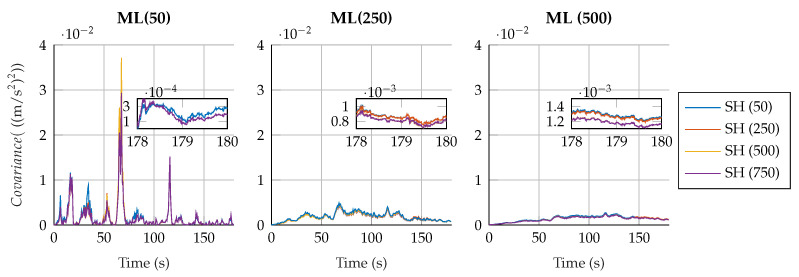
Estimated plant covariance noises under different window sizes in the four-bar linkage with an encoder on the crank.

**Figure 9:**
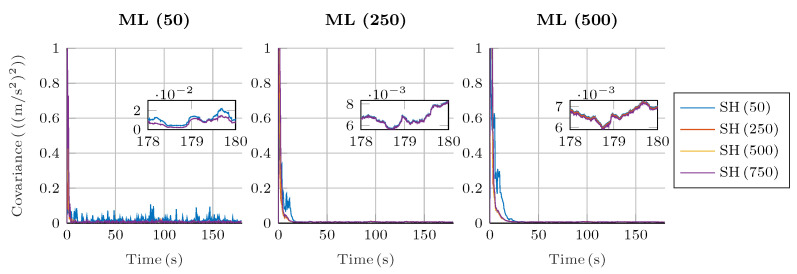
Estimated plant covariance noises under different window sizes in the five-bar linkage with a gyroscope on each crank.

Regarding the estimation of the plant noise, Figure 8 and Figure 9 show the estimated value of σ in the four-bar linkage with an encoder on the crank and the five-bar linkage with a gyroscope on each crank. It can be seen how, despite the window size, the estimation of the plant noise converges to a value of the same order of magnitude. However, the estimation shows more oscillations with small window sizes, while it is more consistent with high window sizes.

It is interesting to see how, for a window size of 50 samples for both algorithms, the fluctuations in the plant noise estimation in the five-bar linkage are strong compared with higher values of window size for the shaping filter. Meanwhile, in the four-bar linkage with an encoder on the crank, the estimated plant noise is similar for the different window sizes employed in the shaping filter. This reflects again the effect of not correcting the autocorrelation of the innovation sequence; if the window size of the shaping filter is small, and the innovation tends to be autocorrelated, the shaping filter will not be able to effectively correct the innovation sequence, thus reducing the overall performance of the filter.

### 3.2. State Estimation

**Figure 10:**
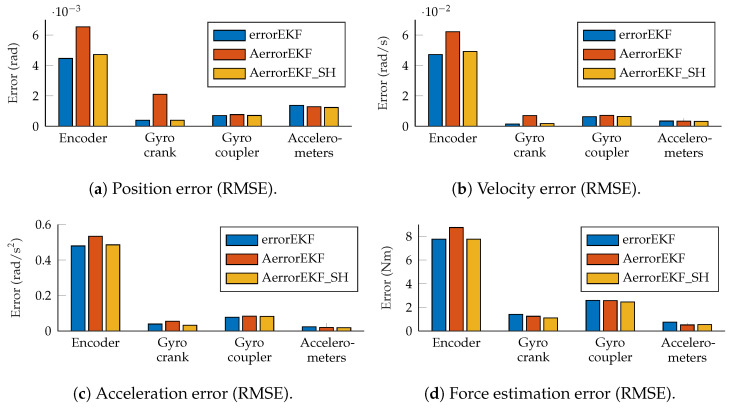
RMS error measured at position, velocity, acceleration and force levels, all evaluated at the crank angle.

The accuracy of the estimations provided by the AerrorEKF_SH is tested using again the the four-bar and five-bar linkage mechanisms (Figure 1) considering the different set of sensors depicted in Figure 2. The window sizes for the plant noise estimation and ψ estimation are of 500 samples for both algorithms. The tests considered were performed using the *three-simulation method*, with the conditions explained in Section 2. The tests were performed with simulations of 180 s, but the first 20 s were not taken into account to let the adaptive methods capture the behavior of the mechanism and adapt to it. The root mean squared error (RMSE) of the test with the four-bar linkage is shown in Figure 10.

These figures show that all the methods perform to a similar level (same order of magnitude). The errorEKF is usually more accurate than the adaptive errorEKF (AerrorEKF), but when the shaping filter is added (AerrorEKF_SH), it reaches about the same accuracy as the errorEKF. However, this errorEKF was hand-tuned, which is a specialized task that requires a long time, especially for complex systems. On the other hand, the adaptive methods only require the selection of a window size, which is much easier, since it is an integer number. Moreover, they can adapt to variable conditions, such as mechanism degradation, or unknown time-varying forces.

**Figure 11:**
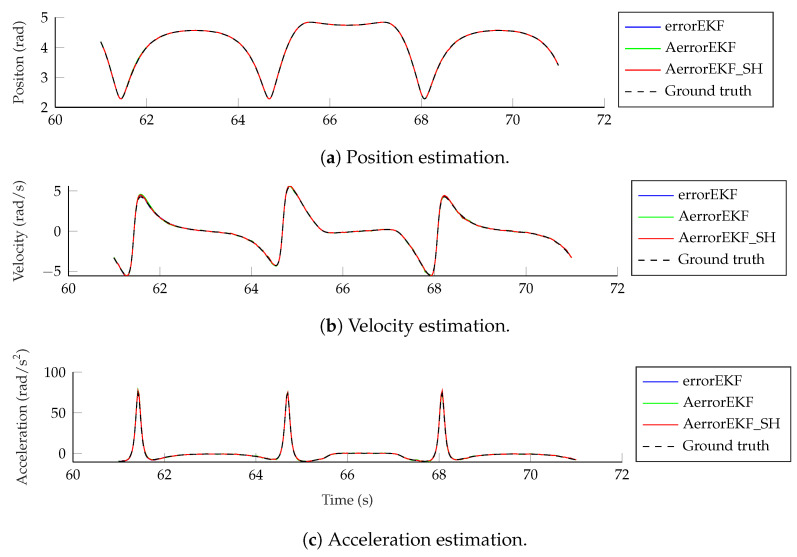
Position, velocity and acceleration estimations using an encoder on the crank.

The estimation accuracy of the kinematic magnitudes is quite high for all the methods and with all the sensors for most applications. The worst estimation is achieved with the encoder, and even in this case, the accuracy of the estimation at position level is better than that of the encoder itself due to the added noise, and thanks to the estimator, accurate estimations of velocity and acceleration are also achievable with only this position sensor. The results from the other sensors considered are better than those of the encoder, and even position estimation is possible for the maneuvers tested using only one angular rate sensor (gyroscope or gyro) on the crank or on the coupler, or a pair of accelerometers on the crank. Position, velocity and acceleration estimations using only the encoder are shown in Figure 11. It can be seen that even with this sensor, which provides the worst estimation accuracy, the estimations are almost indistinguishable from the reference signals.

**Figure 12:**
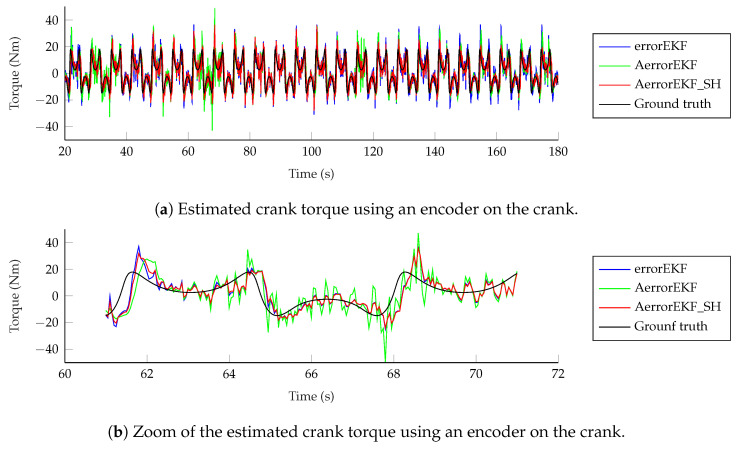
Force estimation with an encoder on the crank.

However, the situation with force estimations is worse. Force estimations achieved with the encoder can be seen in Figure 12. In the upper plot in this figure, the estimation from 20 to 180 s is shown. In the lower plot, just 10 s are shown for the sake of clarity. The three methods behave similarly, with a low-accuracy estimation, and with a noticeable delay when sudden changes in force are required. At this point, it is worth pointing out that there is not actually any torque applied to the crank of the mechanism, but there is a wrong value of the gravity acceleration applied to the mechanism with errors. Since this difference is introduced as a modeling error which is supposedly unknown, the estimator tries to correct it by applying a force on the degrees of freedom which compensates the acceleration error. In this case, since the degree of freedom is an angle, the force is actually a variable torque which aims to compensate for the effect of the gravity error. The reference line provided here is the torque that should be applied to the crank of the model with errors to have the same acceleration as the reference model when both models have the same position and velocity. In other problems, where the designer might know where the error comes from, the method can be modified to apply the correction force in a more realistic way, as shown in [7], where a vehicle is studied, and the correction forces are applied to the tires, instead of being applied to the degrees of freedom. Going back to the plots in Figure 12, it can be seen that the force estimation is not accurate, especially when the force presents a fast change in value, where the estimation acts with a noticeable delay.

**Figure 13:**
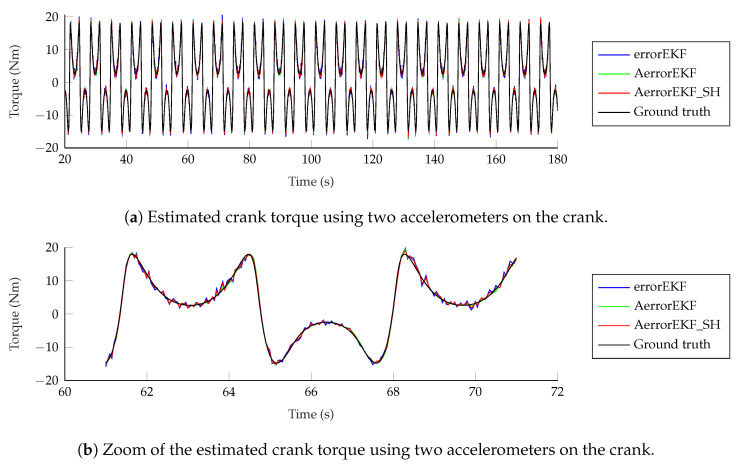
Force estimation with two accelerometers on the crank.

The situation improves with the other sensors. When the gyroscopes are used, the accuracy is improved and the delay is not appreciable any more. Finally, with the accelerometers, the estimation is the best among all the sensor configurations considered in this work. The results of the force estimation with the accelerometers are shown in Figure 13.

**Figure 14:**
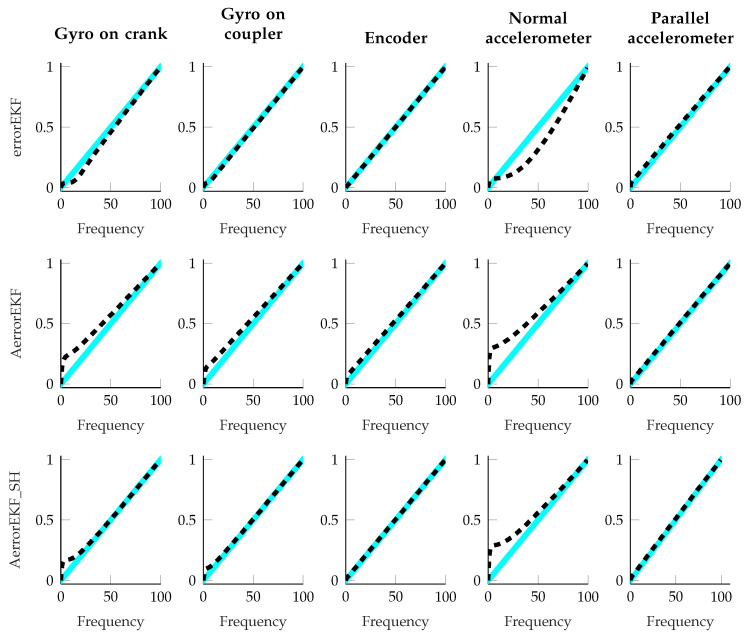
Periodograms of the innovation sequence of all the tests performed with the four-bar linkage. The dashed line represents the periodogram of the innovation, and the blue zone is the 95% confident interval with respect to the ideal periodogram. The rows show the different methods, and the columns show the different sensor sets.

Finally, as an indicator that the estimators are working properly, the sequence of the innovation should resemble white noise, and therefore, the periodogram of the innovation sequence should be a straight line. This information was used to tune the errorEKF by hand, and it is also used to check that the adaptive methods are working properly. The results of all the maneuvers performed with the four-bar linkage are shown in Figure 14. All the methods approximate a straight line quite well, with the exception of the normal accelerometer (the accelerometer installed perpendicular to the crank), whose innovation periodogram is the worst. The trends of the periodograms are very similar for both adaptive methods, but the method with the shaping filter is closer to a straight line in all cases. It is worth pointing out that the plant noise for the errorEKF is constant and it is the same for all the maneuvers, while the adaptive methods vary the plant noise continuously, and thus, it is not constant even within every single test.

**Figure 15:**
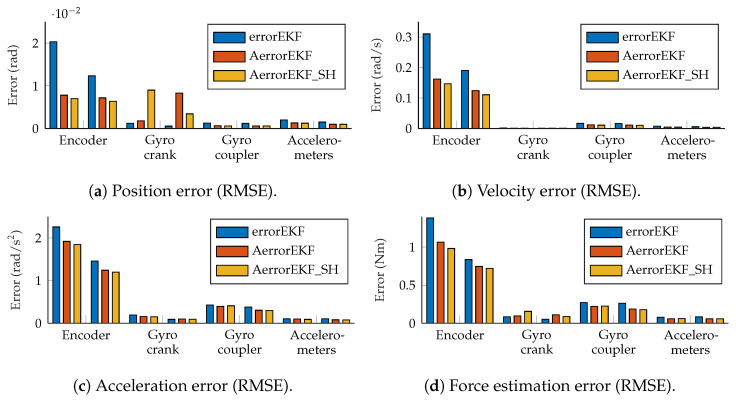
RMS error of the five-bar mechanism measured at position, velocity, acceleration and force levels, all evaluated at the crank angles.

With the five-bar mechanism, most of the results are similar to those obtained with the four-bar mechanism. The RMS errors are shown in Figure 15. The trends are similar to those observed with the four-bar mechanism, with the exception of the position error with gyros on the cranks, which are higher than expected for both adaptive methods. The reason for this behavior can be partially explained because the convergence of the adaptive methods is slower in this particular example, and it is harder for them to compensate for the initial position error. However, even at the final stage of the tests, when all the methods achieved a steady-state solution, the errorEKF still performs slightly better than both of the adaptive methods on both degrees of freedom.

**Figure 16:**
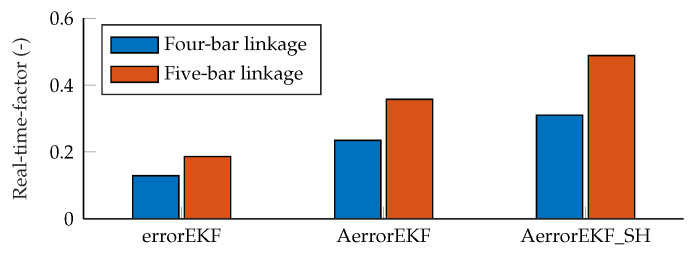
Computational cost of the methods compared in this work.

Regarding the computational cost, a comparison of the different algorithms is shown in Figure 16. However, it must be pointed out that the implementation used in this work was performed in Matlab, whose main focus is not efficiency, and also, the MBDE library used is not intended for efficiency but for flexibility and fast development of new methods. Therefore, the data of computational cost must be taken just as a worst-case scenario. A dedicated program in Matlab might be about twice as fast, and the difference would be greater if a compiled language, such as C++, were used instead. That said, in this case, it can be seen that the increase in computational cost of the five-bar tests with respect to the four-bar ones is 44% for errorEKF, 52% for AerrorEKF, and 57% for AerrorEKF_SH.

Within the same mechanism, the AerrorEKF has a computational cost 1.82 times than that of the errorEKF when tested with the four-bar mechanism, and 1.92 when tested with the five-bar linkage. The AerrorEKF_SH has a computational cost 2.41 times that of errorEKF with the four-bar mechanism, and 2.62 times when the five-bar linkage is evaluated.

## 4. Conclusions

In this work, a new method is proposed in the framework of Kalman filters based on multibody models, known as AerrorEKF_SH. This method includes the characterization of the plant noise, which results in a filter that can react to unknown changes in the system under observation, reducing possible divergences and increasing the accuracy of the estimations. In addition, the characterization of the plant noise simplifies the implementation of the Kalman filter in real applications.

This new method is an improvement over an adaptive Kalman filter already applied to multibody models. In the previous algorithm, the covariance matrix of the plant noise is calculated during the execution of the program, aimed at achieving an innovation sequence which resembles white noise using a maximum likelihood algorithm. However, this is not always possible, because this is only guaranteed when the system is linear, and the noise of both the system and the plant is white and Gaussian. In the case of multibody systems, in many cases, the errors come from incomplete or inaccurate force models, which usually force systematic errors at the plant. For this reason, the design of the errorEKF methods with force estimation assume a random walk model for the unknown forces. But depending on several other factors, such as the type of force error or even the coordinates used, the force error might be constant or not, and therefore, the random walk model might not be the most adequate representation of the force.

For this reason, a shaping filter was added to the adaptive method to whiten the plant noise, and therefore to help the adaptive algorithm to reach its goal. Both the shaping filter and the adaptive method using the maximum likelihood algorithm require a window to extract information from the innovation sequence. The effect of these window sizes was studied, and it was found that a size of 500 samples on both windows works well without increasing the computational cost excessively in the use-cases studied in this work.

The new method was tested in two mechanisms (four-bar and five-bar linkages) using the *three-simulation method*, and compared with the hand-tuned errorEKF and the previous adaptive errorEKF without shaping filter. The new method showed good results, with an accuracy comparable to that of the errorEKF tuned by hand, but with the advantage that no technical knowledge is required to adjust it to other mechanisms, to different maneuvers, to the same mechanism but operating in a degraded condition, or even with variable conditions during the simulation, thus showing several advantages compared to the previous methods described in the literature.

## Figures and Tables

**Table 1:** Properties of the four-bar linkage.

	Crank	Coupler	Rocker	Ground Element
Mass (kg)	2	8	5	-
Length (m)	2	8	5	10

**Table 2:** Properties of the five-bar linkage.

	Left Crank	Left Coupler	Right Coupler	Right Crank	Ground Element
Mass (kg)	3	1	2	3	-
Length (m)	0.5	2.062	3.202	0.5	3

**Table 3:** Characteristics of the sensors considered in this work.

	Encoder	Gyroscope	Accelerometers
Standard deviation	$1.745\times 10^{-2}\;\mathrm{rad}$	$9.839\times 10^{-4}\;\mathrm{rad}/s$	$5.638\times 10^{-2}\;m/s^{2}$
Sampling frequency (Hz)	200	200	200

**Table 4:** Autocorrelation of the innovation for the observations in the encoder in the four-bar linkage for the AerrorEKF_SH with different window sizes and the errorEKF.

	AerrorEKF_SH			errorEKF
ML	50	250	500	
SH				
50	0.0566	0.0038	0.0075	0.0034
250	0.0567	0.0040	0.0080	0.0034
500	0.0559	0.0038	0.0074	0.0034
750	0.0563	0.0038	0.0074	0.0034

**Table 5:** Autocorrelation of the innovation for the observations in the gyroscopes in the five-bar linkage for the AerrorEKF_SH with different window sizes and the errorEKF.

(**a**) First degree of freedom.				
	AerrorEKF_SH			errorEKF
ML	50	250	500	
SH				
50	0.628	0.135	0.116	0.824
250	0.222	0.126	0.113	0.824
500	0.221	0.126	0.110	0.824
750	0.221	0.126	0.110	0.824
(**b**) Second degree of freedom.				
	**AerrorEKF_SH**			**errorEKF**
**ML**	**50**	**250**	**500**	
**SH**				
50	0.187	0.064	0.056	0.443
250	0.109	0.067	0.061	0.443
500	0.108	0.065	0.059	0.443
750	0.108	0.064	0.059	0.443

## Data Availability

The original data presented in the study are openly available in the MBDS/mbde-matlab repository at https://github.com/MBDS/mbde-matlab (accessed on 27 March 2025).
